# Technological Properties and Composition of Enzymatically Modified Cranberry Pomace

**DOI:** 10.3390/foods11152321

**Published:** 2022-08-03

**Authors:** Jolita Jagelaviciute, Loreta Basinskiene, Dalia Cizeikiene, Michail Syrpas

**Affiliations:** 1 Department of Food Science and Technology, Kaunas University of Technology, Radvilenu Rd. 19, LT-50254 Kaunas, Lithuania; jolita.jagelaviciute@ktu.lt (J.J.); dalia.cizeikiene@ktu.lt (D.C.); michail.syrpas@ktu.lt (M.S.); 2 Bioprocess Research Centre, Kaunas University of Technology, Radvilenu Rd. 19, LT-50254 Kaunas, Lithuania

**Keywords:** cranberry pomace, enzymatic hydrolysis, dietary fiber, technological properties

## Abstract

Cranberry pomace obtained after juice production is a good source of dietary fiber and other bioactive compounds. In this study, cranberry pomace was hydrolyzed with Viscozyme^®^ L, Pectinex^®^ Ultra Tropical, Pectinex^®^ Yieldmash Plus, and Celluclast^®^ 1.5L (Novozyme A/S, Denmark). The soluble and insoluble dietary fiber was determined using the Megazyme kit, while the changes in mono-, disaccharide and oligosaccharides’ contents were determined using HPLC-RI; the total phenolic contents were determined by Folin−Ciocalteu’s Assay. Prebiotic activity, using two probiotic strains *Lactobacillus acidophilus* DSM 20079 and *Bifidobacterium animalis* DSM 20105, was investigated. The technological properties, such as hydration and oil retention capacity, were evaluated. The enzymatic treatment increased the yield of short-chain soluble saccharides. The highest oligosaccharide content was obtained using Viscozyme^®^ L and Pectinex^®^ Ultra Tropical. All of the tested extracts of cranberry pomace showed the ability to promote growth of selected probiotic bacteria. The insoluble dietary fiber content decreased in all of the samples, while the soluble dietary fiber increased just in samples hydrolyzed with Celluclast^®^ 1.5L. The highest content of total phenolic compounds was obtained using Viscozyme^®^ L and Pectinex^®^ Ultra Tropical (10.9% and 13.1% higher than control, respectively). The enzymatically treated cranberry pomace exhibited lower oil and water retention capacities in most cases. In contrast, water swelling capacity increased by 23% and 70% in samples treated with Viscozyme^®^ L and Celluclast^®^ 1.5L, respectively. Enzymatically treated cranberry pomace has a different composition and technological properties depending on the enzyme used for hydrolysis and can be used in various novel food products.

## 1. Introduction

Agricultural by-products form a large part of waste, with approximately 88 metric tons per year generated in Europe alone [1]. However, these by-products are considered to be potential sources of various high added-value, bioactive compounds (dietary fibers, pigments, essential minerals, fatty acids, antioxidant polyphenolic compounds, etc.) [2]. Berry pomace (or press cake) obtained after juice pressing usually contains skin, stems, and seed parts [1]. Various fruit and berry products are substantially composed of structural polysaccharides and their related oligosaccharides and these compounds are commonly described as insoluble (IDF) and soluble (SDF) dietary fibers [3]. The dietary fiber in berry pomace is usually composed of pectin, lignin, cellulose, hemicellulose, and inulin [1]. These types of compounds have a potentially significant impact on human health [3], such as a decrease in the risk of hypertension, obesity, diabetes, coronary heart disease, stroke, and certain gastrointestinal disorders [1]. The Food and Drug Administration recommendation for the use of dietary fiber in the diet is approximately 25–35 g per day, and 6 g of it should be SDF. Furthermore, some studies show that 10 g of extra dietary fiber in a diet could help decrease the risk of death caused by coronary heart disease by up to 35% [1,4].

Polyphenols and cell-wall polysaccharides can also positively modulate the profile of the gut microbiota, which may have implications in the prevention against metabolic diseases [5]. Other studies indicate that SDF can be used as a prebiotic [1]. Islam et al. [6] reported that the incorporation of cranberry pomace in the food not only improved the short-term iron and cholesterol levels in the blood serum of broilers, but also increased the levels of beneficial bacterial genera and decreased the undesirable ones. Other studies indicated that the fractions of cranberry oligosaccharide and the xyloglucan and purified cranberry xyloglucan compounds have shown a distinct lack of antimicrobial properties or cytotoxic effects in numerous bacterial and human cell lines [3]. The oligosaccharides extracted from cranberries even shown the ability to modify the biofilm formation of some *E. coli* strains [7]. Liu et al. [8] reported that cranberry oligosaccharides can be used as a carbon source for *Lactobacillus*, however the oligosaccharide metabolization is strain specific. The supplementation of fermented meat with cranberry pomace significantly inactivates *Salmonella* and increases the growth of lactic acid bacteria [9].

An increase in the level of dietary fiber in food products usually negatively influences the products’ texture and color, depending on the properties and level of fiber. However, some studies indicated that enzymatic treatment is practical and a potential modification method to obtain functional food materials with better technological properties from agriculture by-products [10,11]. For example, the enzymatically treated tomato peels have higher contents of lycopene and dietary fiber [10]. The enzymatic treatment can effectively increase the release of antioxidants and phenolic compounds and the fiber composition. However, this is not the case for all of the fruit and berry matrixes; in some cases, enzymatic treatment can reduce the fiber content and increase the free monosaccharides [11]. The treatment of fruit fiber with enzymes of different activities has resulted in structural changes that have altered the technological properties, such as the water-holding and swelling capacities [12].

In this study, cranberry pomace was enzymatically treated with several commercially available enzymes to modify their technological properties and the composition of the dietary fiber. The technological properties were determined, such as water-holding, oil-holding, swelling, emulsion capacities, dietary fiber composition, and prebiotic potential. Most previously published studies indicated cranberry by-products as a good source for isolating bioactive compounds. However, there is a lack of information regarding the effect of enzymatic treatments on cranberry pomace properties. This study provides experimental support for the whole cranberry pomace as a source of dietary fiber and for developing functional and innovative food products.

## 2. Materials and Methods

### 2.1. Cranberry Pomace 

The cranberry pomace was kindly donated by the company “Įvairios sultys” (Kėdainiai, Lithuania). The moisture content was 3.96%. The pomace was ground to a particle size of 0.5 mm and stored at 4 °C.

### 2.2. Proximate Composition Analysis of Cranberry Pomace

The moisture content was determined by drying 1 g of cranberry pomace in an oven at 105 °C to a constant weight, according to the Association of Official Analytical Collaboration (AOAC) Method 925.10-1925. After drying, the final weight of the pomace was subtracted from the initial weight divided by the weight of the sample multiplied by 100 and expressed as g/100 g. The proteins were determined on 1 g of pomace by the Kjeldahl method (N × 6.25), according to the AOAC Method 978.04. The results were expressed as g/100 g of the dry weight sample. The lipids were determined on 1 g of pomace by drying the sample and subsequently the Soxhlet extraction with hexane was performed for 5 h, according to the AOAC Method 948.22. After the extraction, the residues were dried in an oven at 105 °C to a constant weight. The lipid content was calculated from the initial dry weight of pomace by subtracting the final weight of the residues divided by the weight of the dry sample multiplied by 100 and expressed as g/100 g of the dry weight sample. The ash content was determined by charring 2 g of pomace for 30 min, followed by incineration in a muffle furnace at 525 °C for 4 h, according to the AOAC Method 930.05. The residues obtained after incineration were recorded and divided by the weight of the sample weight and multiplied by 100 and expressed as g/100 g of the dry weight sample. The total carbohydrate content was calculated by subtracting the value of the protein, ash, and lipid contents from 100% of the dry weight. The soluble and insoluble dietary fiber were quantified using a Megazyme kit, based on the American Association of Cereal Chemists (AACC) Method 32-07.01 and the AOAC Method 991.43 [13]. One g of pomace (duplicate) was mixed with 40 mL MES-Tris buffer (0.05 M pH 8.2) and hydrolyzed with 50 µL of α-amylase (Megazyme E-BLAAM) at 95 °C for 30 min under constant stirring (120 rpm). After hydrolysis, the temperature was cooled to 60 °C and the sample was hydrolyzed with 100 µL of protease (Megazyme E-BSPRT) at 60 °C for 30 min under constant stirring (120 rpm). Then, the pH was adjusted to 4.1–4.8 with 0.561 N HCl and further hydrolysis was performed with 200 µL of amyloglucosidase (Megazyme E-AMGDF) at 60 °C for 30 min under constant stirring (120 rpm). The suspension was vacuum-filtered in the Fibertec 1023 E equipment (Foss System, Hilleroed, Denmark) through a celite (Megazyme cat. No. G-CELITE) in a bed crucible. The residues were washed with hot distilled water (70 °C), 95% ethanol and acetone, dried and recorded as IDF. The obtained supernatant was mixed with four volumes of 95% ethanol (60 °C) and left to stand at room temperature for 1 h; the SDF was recorded by filtration of the ethanolic suspension in the same conditions as the IDF and washed with 78% ethanol, 95% ethanol, and acetone. The IDF and SDF were calculated by subtracting the protein and ash contents determined in the obtained residues and expressed as g/100 g of the dry weight sample. 

### 2.3. Enzymatic Hydrolysis

The enzymatic hydrolysis of the cranberry pomace was produced using commercially available Novozyme A/S enzymes (Novozyme, Denmark), such as Viscozyme^®^ L (β-glucanases, pectinases, hemicellulases, and xylanases), Pectinex^®^ Ultra Tropical (pectinases, cellulases, hemicellulases, and β-glucanases), Pectinex^®^ Yieldmash Plus (pectinases), and Celluclast^®^ 1.5L (cellulases). The cranberry pomace was mixed with distilled water in a 1:10 ration (2.5 g of pomace with 25 mL of distilled water) and the enzyme was added at various concentration combinations in a range from 0.02 to 0.1 mL/g (control without enzymes). The mixture was incubated at 50 °C with shaking (200 rpm), and various time combinations from 1 to 7 h were used. The independent variable concentration of enzymes and duration of hydrolysis were chosen on the manufacturer’s recommendations, and literature data [14,15]. After the hydrolysis, the enzyme activity was terminated by heating the sample in a 95 °C water bath for 20 min. For the water-soluble fraction yield calculation, the prebiotic activity and the mono-, disaccharides and oligosaccharides analysis, the mixture was cooled at room temperature (20 °C) and centrifugated (8000 rpm, 20 min). The water-soluble part (the resulting supernatant) was collected and freeze-dried (Harvest Right, North Salt Lake, UT, USA). The yield of the water-soluble fraction was determined gravimetrically after freeze-drying. The hydrolyzed cranberry pomace samples used for the determination of the functional properties were collected whole and freeze-dried. All of the freeze-dried samples were kept in a dark place at room temperature.

### 2.4. Mono-, Disaccharides’ and Oligosaccharides’ Analysis by High-Pressure Liquid Chromatography with Refractive Index Detector (HPLC-RI)

For the saccharide analysis, 10 mg of the freeze-dried water-soluble fraction, was dissolved in 1 mL of Millipore water (10 mg/mL). The saccharide analysis was performed as previously described by Syrpas et al. [16]. The total oligosaccharides were recorded as the sum of all of the detected and quantified fractions of oligosaccharides with a degree of polymerization (DP) DP 7–10, DP 5–6, DP4, DP3, while the total mono- and disaccharides were recorded as a sum of the sucrose, glucose, fructose, sugar alcohols, and galacturonic acids. According to the obtained results, the samples enzymatically hydrolyzed for 1 h were selected for further analysis.

### 2.5. In Vitro Assessment of the Prebiotic Activity

The in vitro prebiotic activity of the water-soluble fraction was assessed by evaluating the probiotic bacteria growth stimulation, as described by Moreno-Vilet et al. [17] with some modifications. Two probiotic strains, *Lactobacillus acidophilus* DSM 20079 and *Bifidobacterium animalis* DSM 20105, were used. The probiotic strains were grown in De Man Rogosa and Sharpe (MRS) medium (Biolife, Milan, Italy), supplemented with 0.05% L-cysteine (*w*/*v*) under anaerobic conditions at 37 °C for 18 h. The bacterial inoculants were prepared by centrifugation (5000 g, 10 min, 4 °C) and washing twice with saline solution. After that, the bacterial cell pellets were collected and re-suspended in saline solution, using McFarland standard No. 0.5 (Liofilchem, Waltham, MA, USA) to standardize the bacterial concentrations. The growth of probiotics was evaluated using carbohydrate-free culture media with the same composition as the MRS supplemented with 1% (*w*/*v*) from a different carbohydrate source. The water-soluble fraction obtained after 1 h hydrolysis was used as the carbohydrate source. Glucose was used as the negative control; commercial inulin was used as the positive control; and carbohydrate-free media was used as the blank control. Each tube was inoculated with 1% of the probiotic suspension and incubated for 48 h at 37 °C under anaerobic conditions. The probiotic bacteria counts were determined after 24 and 48 h. The probiotic growth was performed in quadruplet and measured by serial dilutions using the standard plate count technique following the calculation of the number of cells on the agar plate after 72 h incubation at optimal temperature and was expressed as log10 value of CFU/g. The proliferative index (PI) was calculated by the following equation:

(1) PI=average LogA − average LogB

where B is the bacterial count at 0 h (CFU/g) and A is the bacterial count at 24 or 48 h (CFU/g).

### 2.6. Total Phenolic Content (TPC) by Folin−Ciocalteu’s Assay

The TPC of the enzymatically modified cranberry pomace was determined by Folin−Ciocalteu’s Assay according to Singleton et al. [18] with some modifications. The extracts were obtained by mixing 1 g of enzymatically hydrolyzed cranberry pomace with 10 mL of methanol solution (0.1% HCl in methanol (*v*/*v*)) and left overnight in the dark at 4 °C. One ml of the extract solution in methanol was mixed with 5 mL of Folin−Ciocalteu’s reagent (1:9 *v*/*v*) and 4 mL sodium carbonate solution (7.5%). The samples were kept in the dark for 30 min and absorption was measured at 765 nm. The TPC was expressed in mg of gallic acid equivalents (GAE)/g of dry weight of cranberry pomace.

### 2.7. Water Retention Capacity (WRC)

The water retention capacity was performed according to Yu et al. [19] with some modifications. The cranberry pomace (0.2 g) was mixed with distilled water (6 mL) at room temperature and left for 18 h. After that, the sample was centrifugated at 5000 rpm for 20 min. The resulting residues were weighted before and after drying at 105 °C. The WRC was calculated by the following equation:

(2) WRC (g/g)=(Mb−Ma)/Ma

where Mb is the weight of residues before drying (g) and Ma is the weight of residues after drying (g).

### 2.8. Water Swelling Capacity (WSC)

The water-swelling capacity was determined according to Yu et al. [19] with some modifications. The cranberry pomace (0.2 g) was weighted in graduated test tubes and hydrated in 6 mL of distilled water at room temperature for 18 h. The volume of the pomace was recorded before and after hydration; the WSC was calculated by the following equation:

(3) WSC (mL/g)=(V1−V0)/W

where V1 is the volume of pomace after hydration (mL); V0 is the volume of pomace before the hydration; W is the weight of the dry pomace prior to hydration (g).

### 2.9. Oil Retention Capacity (ORC)

The ORC was performed according to Yu et al. [19] with some modifications. The dried pomace (0.2 g) was mixed with 2 g of sunflower oil and left for 1 h at room temperature. After that, the mixture was centrifugated at 300 rpm for 10 min and the supernatant was carefully decanted, and the pellet recovered was weighed. ORC was calculated by the following equation:

(4) ORC (g/g)=(W1−W0)/W0

where W1 is the weight of the pellet (g) and W0 is the weight of the dry sample (g).

### 2.10. Solubility

The yield of soluble material in the pomace was determined, using the supernatant from WRC analysis. The supernatant, after decantation, was dried at 105 °C to a constant weight. The solubility was calculated by the following equation:

(5) Solubility (%)=(M/M0)×100

where M is the weight of dried soluble material (g); M0 is the weight of the dry pomace used for analysis (g).

### 2.11. Emulsion Stability

The emulsion was prepared by mixing the pomace (0.16 g) with sodium phosphate (0.02 M) and citric acid (0.1 M) buffer pH 4, 6, 8 or distilled water (8 g), then the sunflower oil was added (8 g) and emulsified using a rotor–stator homogenizer (IKA^®^ T-25 digital, Ultra-Turrax, Staufen, Germany) for 5 min at 10,000 rpm. The emulsions were transferred into graduated tubes and stored for 3 weeks at 4 °C. The emulsion stability was recorded in terms of phase separation and expressed by the following equation:

(6) Emulsion stability =(VE/VT)×100

where VE is the volume of emulsion; VT is the total volume.

To evaluate the thermal stability of emulsions, the tubes were heated in an 80 °C water bath for 30 min. Then, the tubes were cooled to room temperature and stored for 3 weeks at 4 °C. The thermal stability of the emulsion was recorded and expressed as previously described.

### 2.12. Statistical Analyses

The mean values and standard deviations were calculated using MS Excel 2019 (Microsoft Corp, Albuquerque, NM, USA). The statistical analysis was performed using the Statgraphics Centurion 19 statistical package. One-way analysis of variation (ANOVA), followed by Tukey’s honest significant difference (HSD) test were performed to determine significant differences (*p* < 0.05).

## 3. Results and Discussion

### 3.1. Chemical Composition 

The chemical composition of the cranberry pomace is shown in Table 1. The major component of the pomace is carbohydrates, in which dietary fibers represent 72.67 g/100 g of dry weight (IDF—59.93 g/100 g and SDF—12.74 g/100 g). The dietary fibers obtained from the various berries mainly include: lignin, pectin, cellulose, and hemicellulose [1]. Islam et al. [6] reported a similar amount of SDF (11–12.3 g/100 g) in cranberry pomace, while the protein content was reported as lower (5.75 g/100 g). White et al. [20] reported a similar carbohydrate content in cranberry pomace, but a lower SDF and protein content and a higher IDF and fat content. The chemical composition of the cranberry pomaces varies and is influenced by several conditions, such as the cultivar, ripeness, or processing conditions.

### 3.2. Yield of Water-Soluble Fraction

The enzymatic hydrolysis of the cranberry pomace was performed using four different commercial enzymes. The different times for hydrolysis and different concentrations of enzymes were evaluated (rationale). The yield of the water-soluble fraction obtained after enzymatic hydrolysis is presented in Table 2. As could be expected, the enzymatic hydrolysis increased the water-soluble material content in all of the samples. The extraction yield depended on the composition and concentration of enzymes, and the duration of the hydrolysis. The highest yield of the water-soluble fraction was obtained using Viscozyme^®^ L and Pectinex^®^ Ultra Tropical enzymes. Compared with the yield of the control sample after 1 h of incubation, the lowest increase in the water-soluble fraction was observed with Celluclast^®^ 1.5L (increased by 19.68%), while the highest increase was in the sample hydrolyzed with Pectinex^®^ Ultra Tropical (increased by 93.34%). Previous studies have reported that enzymatic treatment increases the water-soluble fraction yield [10]. Yoon et al. [21] reported an increase in the alcohol-insoluble dietary fiber by prolonging the time of enzyme hydrolysis, while the alcohol-soluble dietary fiber yield did not change significantly after 24 h of enzymatic hydrolysis. However, increasing the enzyme level can reduce hydrolysis time (Table 2). In most cases, there were no significant differences (*p* < 0.05) between the yields of water-soluble fraction obtained using the same enzyme by increasing the enzyme level and decreasing enzymatic hydrolysis time.

### 3.3. Saccharide Analysis

The total oligosaccharides, mono- and disaccharides increased in all of the enzymatically hydrolyzed samples (Table 2). The quantity of oligosaccharides and mono- and disaccharides depended on the type and concentration of the enzyme as well as the duration of hydrolysis. The highest increase in total oligosaccharides after 1 h hydrolysis was obtained in the sample hydrolyzed with Viscozyme^®^ L (3.6 times higher than control), while the lowest increase was determined in the sample hydrolyzed with Pectinex^®^ Yieldmash Plus. Viscozyme^®^ L is composed of β-glucanases, pectinases, hemicellulases, and xylanases. The enzymatic hydrolysis of β-glucan-type hemicellulose polymers in cranberry pomace increases the level of oligosaccharides [3].

Spadoni Andreani et al. [22] reported cranberry extracts after enzymatic hydrolysis with a significantly increased glucose level. The highest mono- and disaccharide content was observed in the sample hydrolyzed with Pectinex^®^ Ultra Tropical. The lowest mono- and disaccharide content was determined in the sample hydrolyzed with Celluclast^®^ 1.5L (1.7 times lower than in the pomaces hydrolyzed with Pectinex^®^ Ultra Tropical) compared with other enzymatically hydrolyzed pomaces. A longer enzymatic hydrolysis increased the mono- and disaccharide content, except for the pomace treated with Pectinex^®^ Yieldmash Plus. However, a lower enzyme concentration was used for the longer hydrolysis. The greater increase in the saccharide content was determined using enzymes, which were mainly pectinases. Viscozyme^®^ L used in this study (0.04 mL/g for 1 h hydrolysis) showed the highest level of total oligosaccharides, while the longer duration of hydrolysis and the higher enzyme concentration decreased the level of the oligosaccharides and increased the level of the mono- and disaccharides. 

For these reasons, the determination of the further functional properties was evaluated in the enzymatically hydrolyzed samples for 1 h.

### 3.4. In Vitro Prebiotic Activity

The growth of the probiotic bacteria in a medium supplemented with different carbohydrate sources is shown in Table 3. Both of the tested probiotic bacteria showed an ability to utilize the carbohydrates from cranberry water-soluble fractions as their carbon sources. The growth of the probiotic bacteria in the medium supplemented with carbohydrates was significantly higher than in the carbohydrate-free media. *L*. *acidophilus* DSM 20079 did not survive in the carbohydrate-free media after 48 h, while *B. animalis* DSM 20105 showed an ability to grow in this media. The cranberry water-soluble fraction after 24 h promoted the growth of *L. acidophilus* DSM 20079 better than glucose or inulin. However, with *L*. *acidophilus* DSM 20079 viability decreased in all of the samples after 48 h, except for the media supplemented with glucose when viability was compared after 24 h, whereas with *B. animalis* DSM 20105, growth increased in all of the samples after 48 h (Table 3). The highest increase was observed in the media supplemented with glucose and inulin. The cranberry water-soluble fraction obtained after treatment with Pectinex^®^ Ultra Tropical promoted the growth of *L*. *acidophilus* DSM 20079 better than the other water-soluble fractions after 24 h. Furthermore, after 48 h, the *L*. *acidophilus* DSM 20079 in this medium showed a better cell viability than in the other media supplemented with cranberry extracts. However in most of the cases, no statistically significant differences were observed (*p* < 0.05). It may be due to a higher concentration of mono- and disaccharides (Table 2) in this extract compared with the other cranberry extracts. The same proliferation effect of prebiotics with a higher sugar content was reported in other studies [23,24]. *L*. *acidophilus* could utilize a variety of carbohydrates, such as mono-, di-, and polysaccharides [25]. The *L. acidophilus* could use oligosaccharides as a carbon source and grow without sugar [26]. All of the cranberry water-soluble fractions had oligosaccharides which may have an impact on the faster proliferation of *L*. *acidophilus* DSM 20079, compared with glucose and inulin. However, the faster utilization of carbohydrates may lead to a decreased viability of the probiotic cells after 48 h.

The extract obtained after treatment with Celluclast^®^ 1.5L promoted the growth of *B. animalis* DSM 20105 after 24 h better than the other extracts, however, in most cases, no statistically significant differences were observed (*p* < 0.05). The *Bifidobacterium* shows a specific preference for prebiotic substrates within the genus, although most of the bacteria could utilize a range of different carbohydrates [27]. The molecular weight, degree of polymerization, and the type of linkage between the comprising units are known to influence the prebiotic activity of carbohydrates [23,28,29,30].

### 3.5. Dietary Fiber and TPC Content after Enzymatic Hydrolysis

The dietary fiber content after enzymatic hydrolysis was evaluated in whole freeze-dried samples hydrolyzed for 1 h (Table 4). The IDF significantly decreased (*p* < 0.05) in all of the samples. The lowest content of IDF was observed in a sample hydrolyzed with Pectinex^®^ Ultra Tropical (13.3% lower than the control). The pectinolytic enzymes increased the plant cell-wall breakdown of the pomace [31]. The content of the SDF decreased significantly (*p* < 0.05) in all of the samples, except for the sample hydrolyzed with Celluclast^®^ 1.5L. The lowest SDF content was observed in the sample treated with Pectinex^®^ Ultra Tropical (84.41% lower than the control) and the highest content was determined in the sample treated with Celluclast^®^ 1.5 L, however, no significant differences were observed (*p* < 0.05) compared to the control. The results indicate that the SDF can be hydrolyzed to smaller fragments and does not precipitate with ethanol. Mrabet et al. [32] reported a similar decrease in the dietary fiber content treated with Viscozyme^®^ L, while Yoon et al. [21] reported an increase in the alcohol-soluble dietary fiber after the enzymatic hydrolysis of carrot pomace. Spadoni Andreani et al. [33] reported an increased yield of oligosaccharides in cranberry carbohydrate extracts after enzymatic treatment with Viscozyme^®^ L and Pectinex^®^ Ultra SPL. The obtained results suggest that Celluclast^®^ 1.5L could be used for increasing the SDF, while Viscozyme^®^ L and Pectinex^®^ Ultra Tropical showed promising results for the production of oligosaccharides.

The TPC of the enzymatically treated cranberry pomace was evaluated (Table 4). The enzymatic hydrolysis increased the TPC in all of the samples, and varied in range from 7.04 to 7.96 mg GAE/g, however, no significant difference (*p* < 0.05) was determined between the control and the pomaces hydrolyzed with Celluclast^®^ 1.5L and Pectinex^®^ Yieldmash Plus. The highest amount of TPC was determined after hydrolysis with Pectinex^®^ Ultra Tropical and Viscozyme^®^ L (13.07 and 10.94% higher than the control, respectively). The results showed that decreasing the total dietary fiber content increases the TPC content. The antioxidants are usually stored in natural cell compartments, so they must be released during digestion to be absorbed in the gut [34]. Gouw et al. [35] reported TPC in dried cranberry pomace of 7.58 mg GAE/g; other studies reported phenolic content of 6 mg/g [20], while Ross et al. [36] reported higher values of TPC in cranberry pomace (~13.55–15.17 mg GAE/g) depending on the drying conditions. The TPC content in the berries depends on many factors, such as cultivar peculiarities, cultivation technologies, region, weather conditions, ripeness, harvesting time, and storage conditions/time [37]. The higher content of TPC was reported in grape pomace (38.7 ± 0.36 mg GAE/g) [38] and blueberry pomace (~17.76–20.82 mg GAE/g) [36]. The phenolic compounds associated with soluble dietary fiber may present different structures, including soluble flavonoids and phenolic acids [34].

### 3.6. Technological Properties

Fiber-rich by-products can be included in food products as low-cost and non-caloric bulking agents to partially replace flour, fat or sugar, as water and oil retention agents, and to improve the stability of emulsions [39]. The technological properties were evaluated of samples hydrolyzed for 1 h (Table 4). The ORC after enzymatic hydrolysis significantly decreased (*p* < 0.05) in all of the samples compared to the control. The lowest ORC was observed in a sample hydrolyzed with Pectinex^®^ Yieldmash Plus; however, a significant difference (*p* < 0.05) between the samples treated with enzyme preparations with pectinases was not observed. Zheng et al. [40] reported a decrease in the coconut cake dietary fiber ORC after enzymatic hydrolysis with cellulase. Oil retention is related mainly to the surface properties, overall charge density, and the constituents’ hydrophilic nature [39]. Enzymatic hydrolysis decreased the total dietary fiber ratio and quantity, which may influence the surface properties of the enzymatically treated pomace. Despite the decreased ORC, all of the pomaces showed good ORC properties compared to the other berry pomaces. Gouw et al. [35] reported a lower ORC of cranberry pomaces of 1.97 g/g; other authors also reported lower ORC values of berry pomaces (blackcurrant, redcurrant, chokeberry, rowanberry, and gooseberry) that ranged from 1.91 to 2.27 g/g [41]. Dietary fiber with a good ability to retain oil can be used for the stabilization of emulsions and high-fat food products [39].

The WSC increased in the samples hydrolyzed with Viscozyme^®^ L and Celluclast^®^ 1.5L (23.33% and 70%, respectively) compared to the control, while treatment with other enzymes decreased the WSC. The enzymatic hydrolysis of cellulose and hemicellulose leads to the exposure of more hydrogen bonds, which influence a higher WSC [40]. However, a high IDF and a low SDF content (especially pectin) have an adverse effect on the WSC [42]. The particle sizes also affect the WSC, and, in most cases, the decrease in the particle size increases the WSC; however, it can also be reduced due to the destruction of the dietary fiber matrix and the links between polysaccharides [43]. Reißner et al. [41] reported higher WSC (5.50–7.09 mL/g) of other berry pomaces (blackcurrant, redcurrant, gooseberry, rowanberry, and chokeberry). Gouw et al. [35] reported a higher WSC of cranberry pomace (5.87 mL/g). A previous study showed that the WSC in most cases increased after enzymatic treatments. However, it depends not only on the kind of fiber or enzyme, but on other conditions used in the treatment [12].

The WRC decreased significantly after enzymatic treatment with most of the enzyme preparations, while enzymatic treatment with Celluclast^®^ 1.5L increased the WRC, however, no significant differences were observed (*p* < 0.05) compared with the control. The fibers consisting mainly of primary cell walls generally have higher water hydration values than the fibers consisting mainly of secondary cell walls [44]. The properties of water hydration decreased after enzymatic hydrolysis with enzymes, the main declared activities being pectinases. Spadoni Andreani et al. [33] indicated that the cranberry pomace cell wall is rich in pectic polysaccharides. The hydration properties of dietary fibers are strongly related to the source of the dietary fiber [39,41].

**Figure 1:**
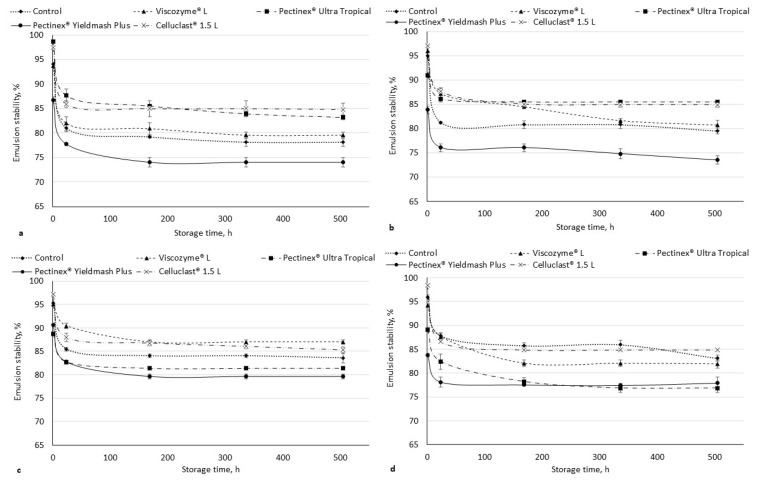
Emulsion stability of enzymatically hydrolyzed cranberry pomaces at pH 3.27 (water) (**a**); 4 (**b**); 6 (**c**); 8 (**d**). Data values are expressed as means with the standard deviation (*n* = 3).

**Figure 2:**
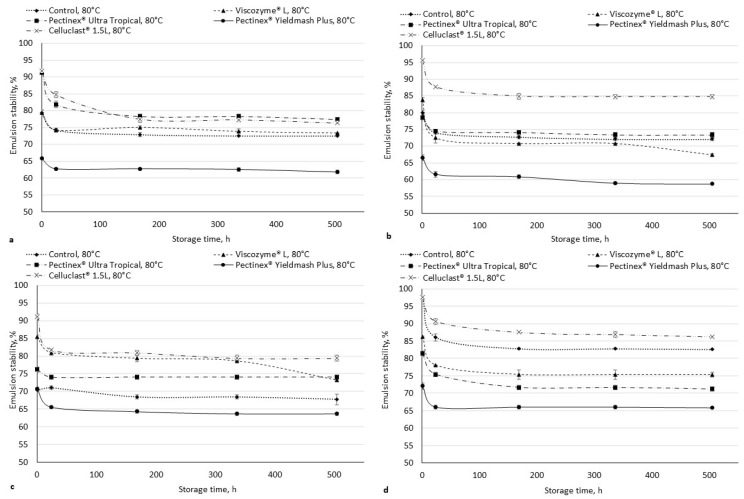
Thermal emulsion stability of enzymatically hydrolyzed cranberry pomaces at pH 3.27 (water) (**a**); 4 (**b**); 6 (**c**); 8 (**d**). Data values are expressed as means with the standard deviation (*n* = 3).

The stability of the emulsion is the ability to maintain the emulsion and its rupture resistance [45]. Kalla-Bertholdt et al. [46] reported that an emulsion prepared with high amounts of SDF has a micelle-like network, leading to a faster initial fat digestion. The emulsion stability of the enzymatically hydrolyzed cranberry pomaces was determined (Figure 1). The stability of the emulsion depended not only on the enzymatically treated pomace, but also on the pH value. This also confirms other studies [47]. The stability of the emulsion decreased during storage in all of the samples, however, in most cases, the stability of the emulsion did not change significantly (*p* < 0.05) during 168 and 504 h of storage (Figure 1). The pomaces hydrolyzed with Pectinex^®^ Yieldmash Plus showed the lowest emulsion stability compared with the other samples. The pomaces hydrolyzed with Pectinex^®^ Ultra Tropical and Celluclact^®^ 1.5 L showed the highest emulsion stability in water (pH 3.27) and pH 4 buffer solution, while, at pH 6, better emulsion stability was shown in the pomaces hydrolyzed with Viscozyme^®^ L and Celluclast^®^ 1.5L. The control sample and the sample hydrolyzed with Celluclast^®^ 1.5L showed a higher stability of the emulsion in a pH 8 buffer compared to the other enzymatically treated pomaces. The thermal stability of the emulsions was lower (Figure 2). The lowest thermal stability in all of the cases was observed using pomaces hydrolyzed with Pectinex^®^ Yieldmash Plus. The highest stability was obtained in most of the cases using pomace hydrolyzed with Celluclast^®^ 1.5L. Huc-Mathis et al. [48] reported that IDF helps maintain the stability of the emulsions through the Pickering mechanism and/or network formation in the continuous phase, probably favored by the stabilization of the proteins and the pectins in the soluble fraction.

## 4. Conclusions

Cranberry pomace is a good source of dietary fiber, containing 59.93 g/100 g (dry weight) of IDF and 12.74 g/100 g of SDF. The enzymatic hydrolysis changed the technological properties of cranberry pomace, the ratio of SDF and IDF, and their quantities. The pomace treated with Celluclast^®^ 1.5L resulted in the highest SDF content and an increase in the WSC and WRC. The highest amount of oligosaccharides was obtained with Viscozyme^®^ L, while hydrolysis with Pectinex^®^ Ultra Tropical resulted in the highest amount of mono- and disaccharides and TPC content. The pomace treated with these enzymes can be used to enhance products with oligosaccharides and phenolic compounds. All of the tested water-soluble fractions showed prebiotic activity and enhanced the growth of *Lactobacillus acidophilus* DSM 20079 and *Bifidobacterium animalis* DSM 20105 after 24 h of fermentation, while viability after 48 h of fermentation was strain and carbon source-dependent. The ORC after the enzymatic hydrolysis significantly decreased (*p* < 0.05) in all of the samples. However, all of the pomaces showed good ORC properties and could be used to stabilize high-fat foods. The highest emulsion stability was obtained in most cases using the pomace hydrolyzed with Celluclast^®^ 1.5L. The enzymatic hydrolysis using different enzymes gave cranberry pomaces with different compositions and technological properties, which could be used in developing different novel food products. Therefore, enzymatic hydrolysis can open new market perspectives for the application of cranberry pomace as a new, cheap and valuable ingredient to improve the nutritional values and technological properties of food products.

## Figures and Tables

**Table 1:** Chemical composition of cranberry pomace.

Parameter	Content (g/100 g)
Moisture	5.57 ± 0.11
Total fat ^a^	9.83 ± 0.46
Ash ^a^	0.96 ± 0.04
Protein ^a^	7.40 ± 0.06
Carbohydrate ^b^	81.81
Insoluble dietary fiber ^a^	59.93 ± 1.46
Soluble dietary fiber ^a^	12.74 ± 0.09

^a^ Expressed as g/100 g of dry weight; ^b^ Carbohydrate content was determined by subtracting the value of protein, ash, and lipid content from 100% of dry weight. Data values are expressed as means with the standard deviation (*n* = 3).

**Table 2:** Water-soluble fraction yield and saccharide composition of cranberry pomace after enzymatic hydrolysis.

Time (h)	E/S Ratio (mL/g)	Enzyme	Water-Soluble Fraction Yield, %	Total Oligosaccharides, mg/g DW	Total Mono- and Disaccharides, mg/g DW
1	0.04	Viscozyme^®^ L	25.56 ± 1.33 ^e^	71.6 ± 4.4 ^b^	178.5 ± 8.9 ^abcd^
5.5	0.1	Viscozyme^®^ L	27.98 ± 0.20 ^e^	61.3 ± 2.1 ^b^	222.9 ± 4.3 ^d^
1	0.1	Pectinex^®^ Yieldmash Plus	21.50 ± 0.78 ^d^	21.2 ± 0.6 ^a^	197.0 ± 0.3 ^bcd^
7	0.02	Pectinex^®^ Yieldmash Plus	19.95 ± 0.23 ^cd^	16.3 ± 3.9 ^a^	173.7 ± 11.4 ^abcd^
1	0.08	Pectinex^®^ Ultra Tropical	27.34 ± 1.56 ^e^	59.0 ± 6.0 ^b^	211.9 ± 24.3 ^cd^
4	0.06	Pectinex^®^ Ultra Tropical	27.41 ± 0.71 ^e^	56.4 ± 4.3 ^b^	216.3 ± 12.7 ^cd^
1	0.02	Celluclast^®^ 1.5L	18.50 ± 1.35 ^bc^	32.2 ± 3.3 ^a^	123.3 ± 14.8 ^ab^
7	0.1	Celluclast^®^ 1.5L	21.74 ± 0.71 ^d^	56.5 ± 3.1 ^b^	150.2 ± 9.0 ^abcd^
1	-	-	14.12 ± 0.34 ^a^	19.9 ± 2.2 ^a^	108.9 ± 2.5 ^a^
4	-	-	16.14 ± 0.14 ^ab^	18.9 ± 2.5 ^a^	118.9 ± 7.5 ^a^
5.5	-	-	16.56 ± 0.68 ^abc^	20.9 ± 2.9 ^a^	140.4 ± 20.9 ^abc^
7	-	-	17.08 ± 0.17 ^abc^	22.0 ± 2.9 ^a^	143.3 ± 21.9 ^abc^

E/S—enzyme/substrate (pomace) ratio. - without enzyme (control samples). Data values are expressed as means with the standard deviation (*n* = 3). Values in one column followed by the same letter are not significantly different (*p* < 0.05).

**Table 3:** Growth of probiotics in medium supplemented with different carbohydrate source.

Prebiotics	*Lactobacillus acidophilus* DSM 20079					*Bifidobacterium animalis* DSM 20105				
	0 h	24 h		48 h		0 h	24 h		48 h	
	Log CFU/g		PI	Log CFU/g	PI	Log CFU/g		PI	Log CFU/g	PI
Carbohydrate-free MRS	5.03 ± 0.07 ^a^	4.27 ± 0.13 ^a^	−0.66	- ^a^	−5.03	5.03 ± 0.05 ^a^	6.64 ± 0.09 ^a^	1.61	6.45 ± 0.03 ^a^	1.42
Glucose	4.78 ± 0.20 ^a^	7.31 ± 0.22 ^bc^	2.53	7.62 ± 0.08 ^e^	2.84	5.28 ± 0.05 ^b^	7.99 ± 0.22 ^b^	2.71	9.00 ± 0.41 ^cd^	3.72
Inulin	4.81 ± 0.23 ^a^	7.11 ± 0.25 ^b^	2.29	5.52 ± 0.57 ^b^	0.71	5.28 ± 0.08 ^b^	8.36 ± 0.25 ^bc^	3.08	9.07 ± 0.12 ^d^	3.78
Control	4.77 ± 0.06 ^a^	7.72 ± 0.10 ^de^	2.95	5.86 ± 0.06 ^bc^	1.09	5.52 ± 0.09 ^c^	8.03 ± 0.52 ^b^	2.51	8.83 ± 0.12 ^cd^	3.30
Viscozyme^®^ L	4.79 ± 0.09 ^a^	7.69 ± 0.11 ^cde^	2.90	6.21 ± 0.04 ^cd^	1.41	5.60 ± 0.06 ^c^	8.37 ± 0.13 ^bc^	2.77	8.37 ± 0.05 ^b^	2.77
Pectinex^®^ Ultra Tropical	4.72 ± 0.07 ^a^	7.84 ± 0.23 ^e^	3.13	6.45 ± 0.14 ^d^	1.73	5.58 ± 0.05 ^c^	8.40 ± 0.02 ^bc^	2.81	8.63 ± 0.05 ^bc^	3.04
Pectinex^®^ Yieldmash Plus	5.00 ± 0.01 ^a^	7.53 ± 0.14 ^cde^	2.53	5.81 ± 0.19 ^bc^	0.82	5.52 ± 0.09 ^ab^	8.03 ± 0.52 ^bc^	2.51	8.77 ± 0.19 ^bcd^	3.24
Celluclast^®^ 1.5L	4.74 ± 0.06 ^a^	7.44 ± 0.15 ^cd^	2.70	5.69 ± 0.09 ^b^	0.95	5.62 ± 0.09 ^c^	8.66 ± 0.03 ^c^	3.04	8.93 ± 0.07 ^cd^	3.31

- No growth; data values are expressed as means with the standard deviation (*n* = 4). Values in one column followed by the same letter are not significantly different (*p* < 0.05).

**Table 4:** Dietary fiber, TPC content and technological properties of enzymatically treated cranberry pomace.

Sample	Time (h)	E/S Ratio (mL/g)	IDF, g/100 g	SDF, g/100 g	ORC, g/g	WSC, mL/g	WRC, g/g	Solubility, %	TPC, mg of GAE/g
Control	1	-	62.48 ± 0.46 ^d^	11.23 ± 0.84 ^c^	6.50 ± 0.08 ^a^	1.80 ± 0.3 ^a^	10.59 ± 0.20 ^a^	15.4 ± 0.9 ^a^	7.04 ± 0.41 ^a^
Viscozyme^®^ L	1	0.04	56.46 ± 0.15 ^ab^	2.64 ± 0.26 ^a^	4.37 ± 0.25 ^b^	2.22 ± 0.01 ^b^	8.58 ± 0.24 ^b^	27.5 ± 1.3 ^b^	7.81 ± 0.36 ^b^
Pectinex^®^ Yieldmash Plus	1	0.1	58.48 ± 0.44 ^bc^	5.44 ± 0.30 ^b^	3.99 ± 0.13 ^b^	1.65 ± 0.01 ^a^	7.25 ± 0.22 ^c^	24.0 ± 0.6 ^c^	7.42 ± 0.24 ^ab^
Pectinex^®^ Ultra Tropical	1	0.08	54.17 ± 1.38 ^a^	1.75 ± 0.29 ^a^	4.11 ± 0.16 ^b^	1.57 ± 0.11 ^a^	8.10 ± 0.43 ^b^	29.6 ± 1.2 ^d^	7.96 ± 0.29 ^b^
Celluclast^®^ 1.5 L	1	0.02	60.52 ± 0.17 ^cd^	11.90 ± 0.53 ^c^	5.80 ± 0.37 ^c^	3.06 ± 0.16 ^c^	11.02 ± 0.24 ^a^	20.3 ± 1.0 ^e^	7.06 ± 0.28 ^a^

Data values are expressed as means with the standard deviation of three replicates for SDF, IDF, ORC, WRC, WSC, solubility and of six replicates for TPC. Values in one column followed by the same letter are not significantly different (*p* < 0.05).

## Data Availability

The data presented in this study are available in the article.
